# Supplementary material for: Targeting of eIF6-driven translation induces a metabolic rewiring that reduces NAFLD and the consequent evolution to hepatocellular carcinoma
Source: Nat Commun. 2021 Aug 12;12:4878. doi: 10.1038/s41467-021-25195-1 (PMC8361022; doi:10.1038/s41467-021-25195-1)
Supplement: Supplementary file 1 — Supplementary Information [file 41467_2021_25195_MOESM1_ESM.pdf]

SUPPLEMENTARY INFORMATION

This file contains Supplementary Methods, Supplementary Figures 1-8 and Supplementary Tables 1-3; Supplementary Tables 2 and 3 are provided also as Supplementary Data Files under the section Source Data, Excel File, Human Data.

Supplementary Methods

Primer list

Syber Green Probes

Gene		Primers
**Hmgcr**	FW	5'-TGAGATCCGGAGGATCCAAG-3
	RW	5'-CAGATCTTGTTGTTGCCGGTG-3'
**Mogat1**	FW	5'-TTGACCCATGGTGCCAGTTT-3'
	RW	5'-GTGGCAAGGCTACTCCCATT-3'
**Mvd**	FW	5'-CGAATCTCTCTCAGGCAGGC-3'
	RW	5'GCACCAGGACCAGCTAAAAAC-3'
**Gpam**	FW	5'-TGCAGACGCCGCTGGGCC-3'
	RW	5'-GAACCCACAGTCAACCCAGT-3'
**Ppara**	FW	5'-TCCACGAAGCCTACCTGAAGAA-3'
	RW	5'-GACAAAAGGCGG GTTGTTGC-3'
**Col3a1**	FW	5'-TGACTGTCCCACGTAAGCAC-3'
	RW	5'-GAGGG CCATAGCTGAACTGA-3'
**Col4a1**	FW	5'-GAACTGGGCTGCATTTCCCC-3'
	RW	5'-CCACCACAATCGCCCTTCG-3'
**Ndufs4**	FW	5'CCATCCAGGTTGTTGAGCAC-3'
	RW	5'-GCATGTTATTGCGAGCAGGA-3'
**Uqcrh**	FW	5'- GACCCCAAAGAGGAAGAAGAGG-3'
	RW	5'-CTTCTGTCTGTGACCGGGAA-3'
**Cox5b**	FW	5'-GTGGGCTGCATCTGT GAAGA-3'
	RW	5'-TGGGGCACCAGCTTGTAATG-3'
**Atp5k**	FW	5'-GAGAGGAGAATAGCAGCGGAG-3'
	RW	5'-CTGTCATCTTGAGCTTCCGCC-3'
**Atp5E**	FW	5'-CTACTCT GAAGCGACCCAGC-3'
	RW	5'-GGGAAAACCGGATGTAGCTGA-3'
**Plin1**	FW	5'- ACGACCAGACAGACACAGAG-3'
	RW	5'-GGCTGTAACCTCTCTGAGCA-3'
**Plin2**	FW	5'- TCCTCACATCTAGCAAGG-3'

	RW	5'-ACCTCGGTAGACTGAGGATA-3'
**Adpn**	FW	5'- GCCGAGCCAAGGTGTGAG-3'
	RW	5'-AAAAGTGCAACCCTGTGCATC-3'
**Vcl**	FW	5'- TGGTCTAGCAAGGGCAATGA-3'
	RW	5'-GGCGATATCCTTGGCACACT-3'
**HK2**	FW	5'-CTCGGTTTCTCTATTTGGCCC-3'
	RW	5'-TGGTAGAGATACTGGTCAACCTTC-3'
**Anxa2**	FW	5'-GAGGCTCTCAGCGATACGTG-3
	RW	5'-CACATTGCTGCGGTTTGTCA-3'
**Saa2**	FW	5'- GACACCAGCAGGATGAAGCTA-3'
	RW	5'- ATGTCTCCAGCCCCTTGGAA-3'
**Coroc1**	FW	5'- CAGGAGATTGTGGCGGAGAA-3'
	RW	5'-TAGTGAAGACGTTGCCGTCC-3'
**rpS18**	FW	5'-ATGGGAASGTACAGCCAGGTTC-3'
	RW	5'-CAGTGGTCTTGGTGTGCTGA-3'
**βActin**	FW	5'-GACCTCTATGCCAACACAGTGC-3'
	RW	5'-CCTGCTTGCTGATCCACATCTG3'

TaqMan Probes

**Gene**	**Probe**	
**eIF6**	Mm00550245_m1	
**YY1**	Mm00456392_m1	
**C/EBPB**	Mm00843434_S1	
**PPARG**	Mm00440940_m1	
**Rpl36**	Mm07297453_g1	
**Rps15**	Mm02342443_g1	

Supplementary Figures

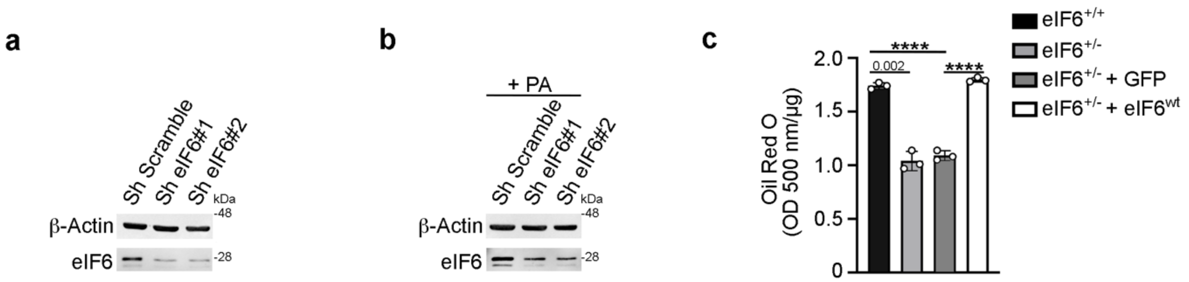

Supplementary Figure 1

Supplementary Fig.1| eIF6 drives lipid synthesis in a cell autonomous fashion

a, Western Blotting of eIF6 levels in indicated AML12 cell lines. Same cells as in Fig. 2d. **b**, Representative Western Blotting on Palmitate-treated ALM12 cells. Same cells as in Fig. 2f. **c**, Quantification of Oil Red O content in EMSC cells from eIF6$^{+/+}$ and eIF6$^{+/-}$ mice. Reconstitution of eIF6 protein levels in eIF6$^{+/-}$ EMSC adipocytes rescued lipid accumulation. GFP transduction was used as control. Data are represented as means ± SD. Two-tailed T-test. Data are provided as a Source Data File.

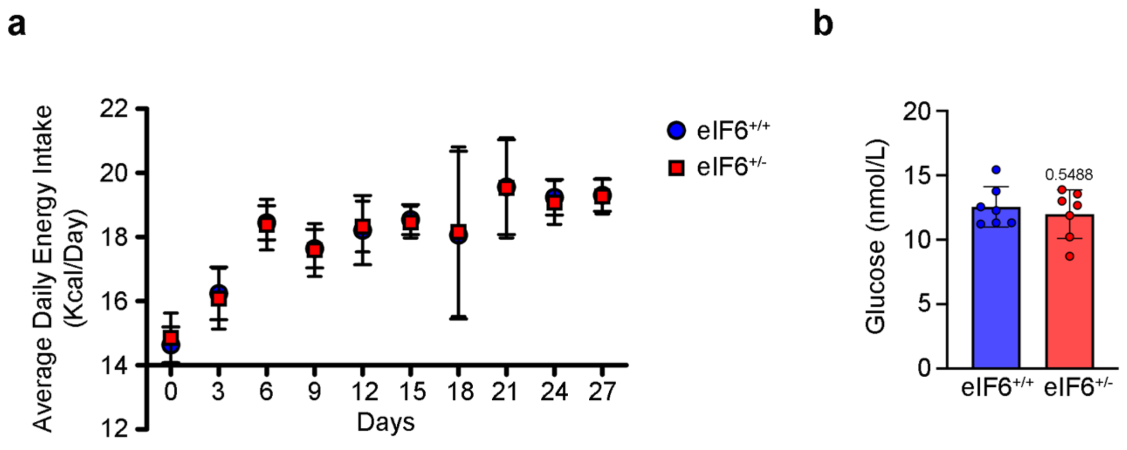

Supplementary Figure 2

Supplementary Fig.2| Food intake and basal glycemia are not affected by eIF6 haploinsufficiency

a, Cumulative food intake, expressed as daily caloric intake, was measured at the indicated time points. No difference between eIF6$^{+/+}$ and eIF6$^{+/-}$ mice was observed. n=7 mice for genotype. Data are represented as means ± SD. **b,** Glucose measurement in the blood of eIF6$^{+/+}$ and eIF6$^{+/-}$. Data are represented as means ± SD. n=7 mice for genotype. Data are provided as a Source Data File.

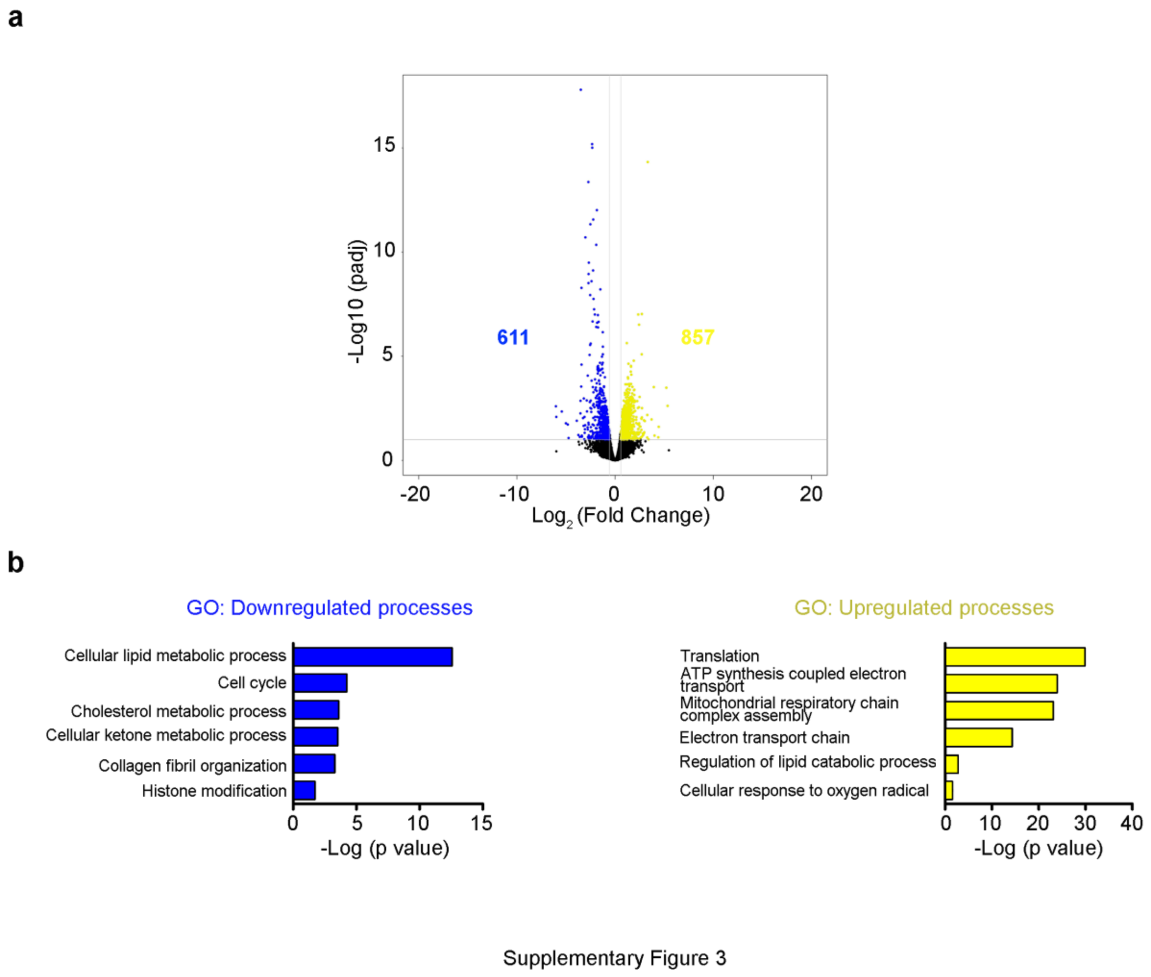

Supplementary Fig.3| RNA-Seq analysis of eIF6$^{+/-}$ versus eIF6$^{+/+}$ livers

a, V0lcano plot shows significantly up-(yellow) and down-(blue) regulated genes from RNA-Seq data. **b,** GO analysis identifies a reduction in lipid biosynthesis, response to insulin and cholesterol metabolic process pathways in eIF6$^{+/-}$ livers under HFD regimen, and additional effects on cell cycle and collagen fibril organization. Up-regulated GO of mitochondrial processes are also shown. Data are provided as a Source Data File.

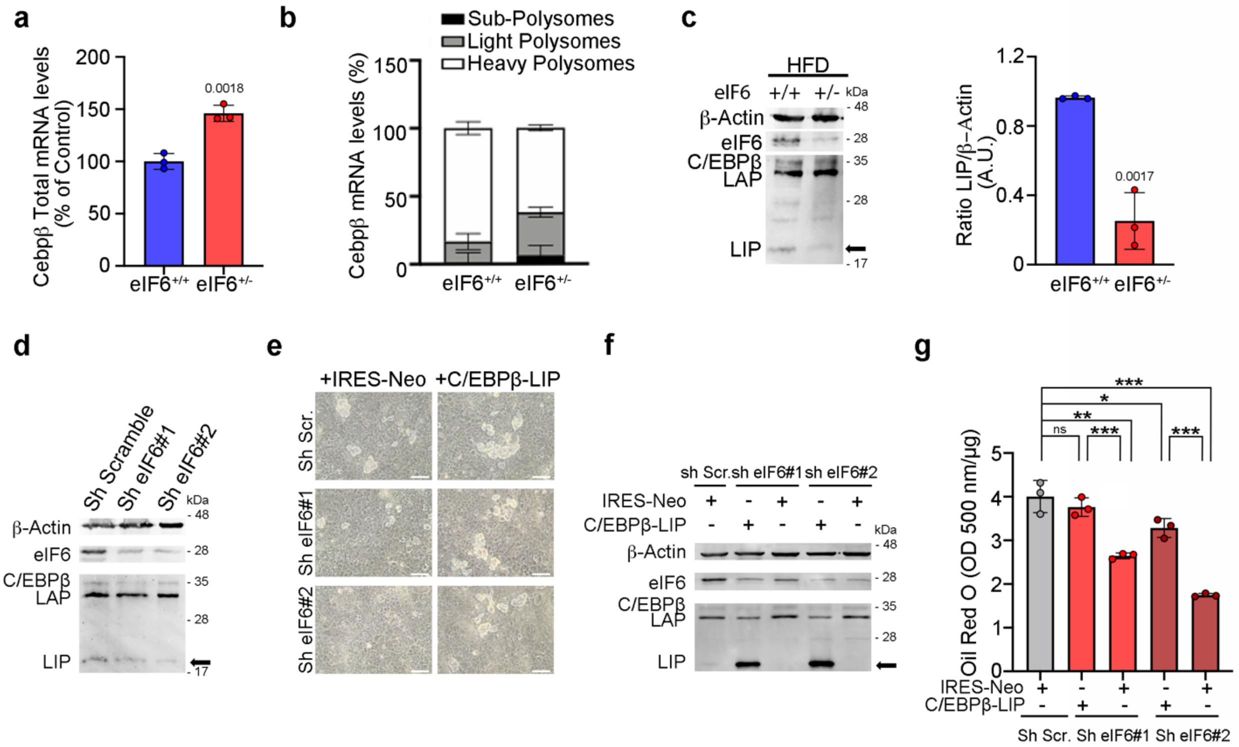

h

TFs	Fc	Pvalue	GO
Cers6	-1.27	0.0001	Lipid biosynthetic process (GO: 0008610)
Nr2c2	-1.54	0.0001	Lipogenesis and lipid metabolic process (GO: 0006629)
Hnf1α	-1.27	0.0017	Regulation of lipid metabolic process (GO: 0019216)
Pparγ	-0.85	0.0049	Lipogenesis and lipid metabolic process (GO: 0006629)
Nr1I3	-0.84	0.0064	Lipogenesis and lipid metabolic process (GO: 0006629)
Pparα	-1.11	0.0078	Lipogenesis and lipid metabolic process (GO: 0006629)

Supplementary Figure 4

Supplementary Fig.4| C/EBPβ is translationally regulated by eIF6

a, Real time PCR analysis of C/EBPβ mRNA in eIF6$^{+/+}$ and eIF6$^{+/-}$ livers. n=3 for genotype. Data are represented as means ± SD. Two-tailed T-test. **b,** Real-time PCR analysis of C/EBPβ mRNA on liver polysome fractions reveals that its association with actively translating ribosomes (Heavy polysomes, white box) is decreased in eIF6$^{+/-}$ livers, compared to wild-type controls. n=3 for genotype. **c,** Left: representative Western Blotting analysis shows that C/EBPβ-LIP protein expression level is decreased in eIF6$^{+/-}$ livers compared to wt ones. Right: densitometric analysis of C/EBPβ-LIP expression normalized on β-Actin. Data are expressed as Arbitrary Unit (n=3 per genotype). Data are represented as means ± SD. Two-tailed T-test. **d**, C/EBPβ-LIP protein expression level is decreased in eIF6 silenced AML12 cells. **e**, Representative images of AML12 transduced with C/EBPβ-LIP vector and IRES-Neo vector as control: lipid droplets accumulation are increased upon C/EBPβ-LIP overexpression. Scale bar = 100 μm. **f**, Western Blotting analysis shows that C/EBPβ-LIP protein (arrow) is overexpressed in AML12 transduced with C/EBPβ-LIP vector. **g**, Quantification of Oil Red O staining: lipid droplets accumulation is restored in C/EBPβ-LIP overexpressed AML12 cells. Data are represented as percentage of control (IRES-Neo-ShScramble) (n=3). Data are represented as means ± SD. *p value≤0.05, **p value≤0.01, ***p value≤0.001. **h,** Transcription factors, targets of C/EBPβ[49] involved in lipogenesis and lipid metabolic processes are down-regulated at the transcriptional level, in eIF6$^{+/-}$ livers. Their relative Fold Change, p values and GO Terms are reported. Data are provided as a Source Data File.

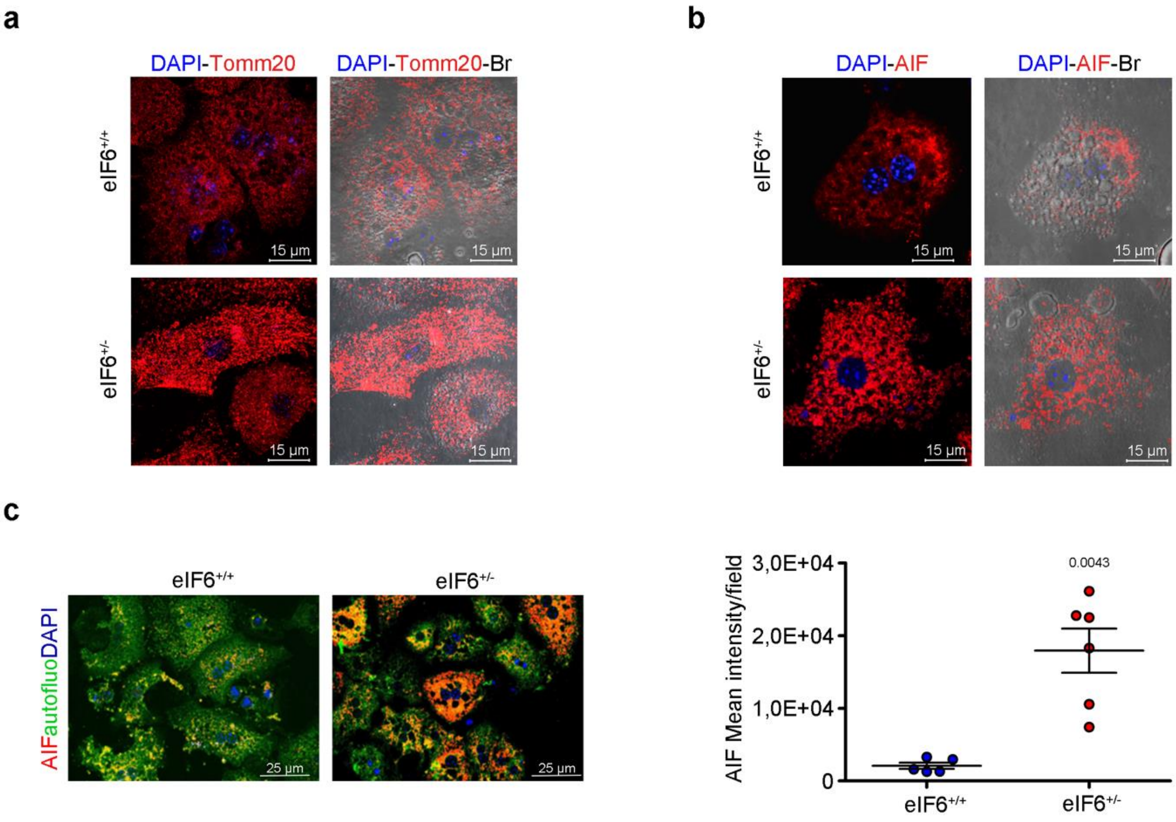

Supplementary Figure 5

Supplementary Fig.5| Mitochondrial network is more consistent in eIF6$^{+/-}$ primary hepatocytes

a, Representative mitochondrial staining with anti-Tomm20 antibody shows higher Tomm20 intensity in eIF6$^{+/-}$ primary hepatocytes compared to wild-type ones. Bright field images were merged with immunostaining. Scale bars are indicated. **b,** Representative bright field images merged with immunofluorescence staining for mitochondrial Aif show higher Aif intensity in eIF6$^{+/-}$ primary hepatocytes compared to wild-type ones. Scale bars are indicated. **c**, Right: Representative images of immunostaining with anti-Aif antibody in eIF6$^{+/+}$ and eIF6$^{+/-}$ primary hepatocytes. Lack of nuclear staining indicates lack of apoptosis. Autofluorescence (green) of hepatocytes is used to visualize primary cells. Scale bars are indicated. Left: quantitation of Aif fluorescence intensity shows that Aif levels are higher in eIF6$^{+/-}$ primary hepatocytes compared to wild-type cells. Horizontal lines represent the mean ± SEM. Mann Whitney test. Data are provided as a Source Data File.

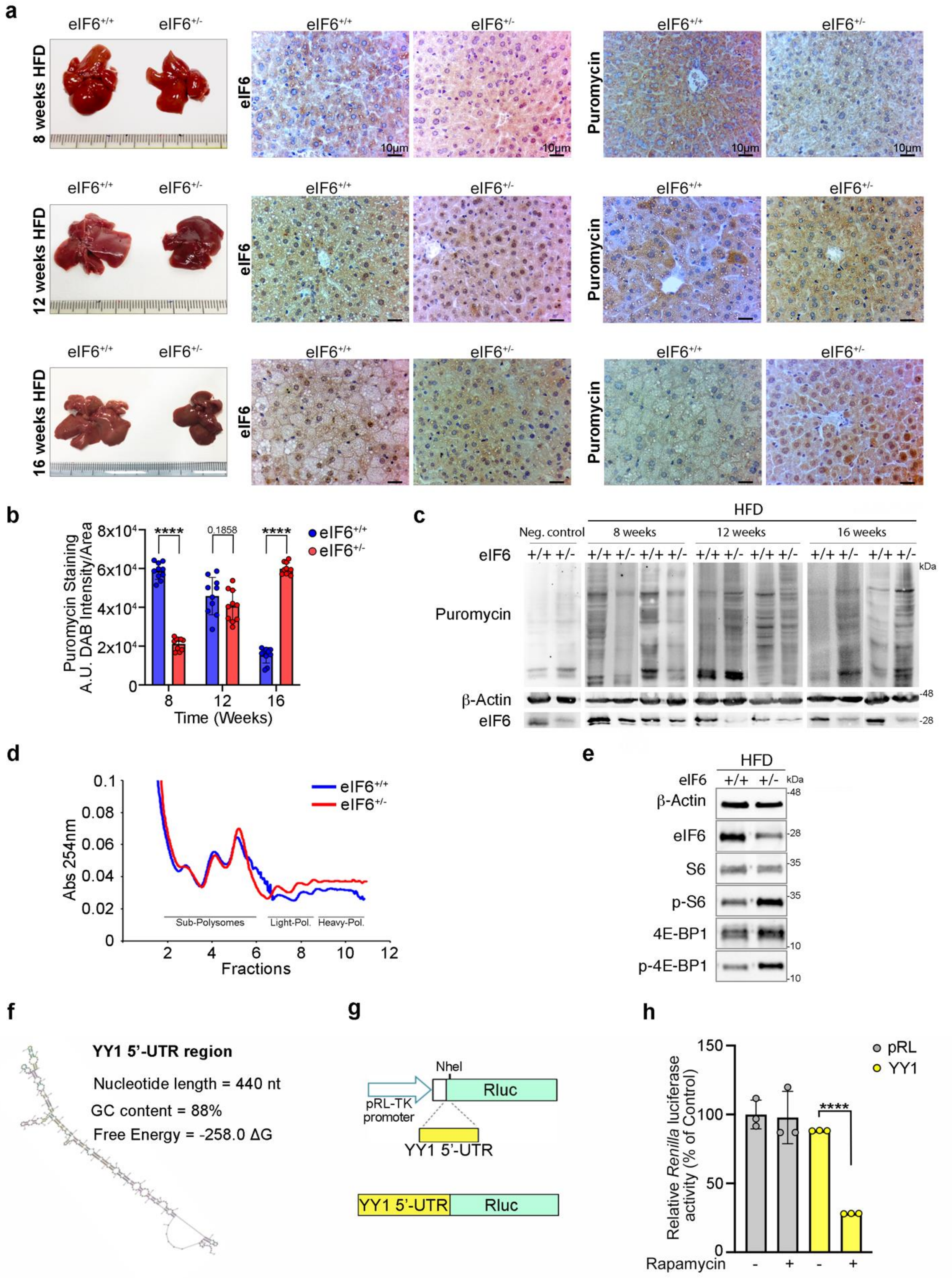

Supplementary Figure 6

Supplementary Fig.6| mTORc1 activation controls YY1 induction at the translational level

a, Gross appearance of livers (left) and representative eIF6 and Puromycin IHC staining on liver sections (right). Mice were sacrificed after 8, 12 and 16 weeks of HFD regimen. Scale bars are indicated. **b**, Quantification of Puromycin labelling at indicated times. N=10. Data are represented as mean ± SD. Two-tailed T-test. **c**, Representative Western Blotting of Puromycin incorporation in $eIF6^{+/+}$ and $eIF6^{+/-}$ livers at indicated time points. Paired littermates animals are shown in each time point. β-actin was used as loading controls. eIF6 levels in heterozygous mice are always reduced in paired littermates. **d**, Representative liver polysomal profile of $eIF6^{+/+}$ and $eIF6^{+/-}$ livers after 16 weeks of HFD regimen. Note that polysome peaks of $eIF6^{+/-}$ livers are slightly increased, but 80S is sharper, consistent with loss of eIF6 antiassociation activity. Peaks aligned to free 40S and 60S show no ribosomal unbalance. Sub-Polysomal and Polysomal fractions are indicated. The experiment was repeated at least three times. **e,** Representative Western Blotting analysis indicates that mTORc1 kinase downstream substrates are more phosphorylated in $eIF6^{+/-}$ livers: rpS6 and 4EBP1 phosphorylation are increased in $eIF6^{+/-}$ samples compared to wt. **f,** Predicted secondary structure of the 5'UTR sequence of YY1 mRNA. Nucleotide length, GC content and Free Energy are reported. **g,** YY1 5' UTR was cloned upstream of the *Renilla* luciferase reporter vector, as shown in the scheme. **h,** *Renilla* luciferase activity of pRL and pRL 5' UTR YY1 constructs in the absence or presence of rapamycin, mTORC1 inhibitor, were measured. n=3 independent experiments. Data are represented as means ± SD. N=10. Two-tailed T-test. The 5'UTR of YY1 mRNA confers sensitivity of translation to mTORC1 inhibition. Data are provided as a Source Data File.

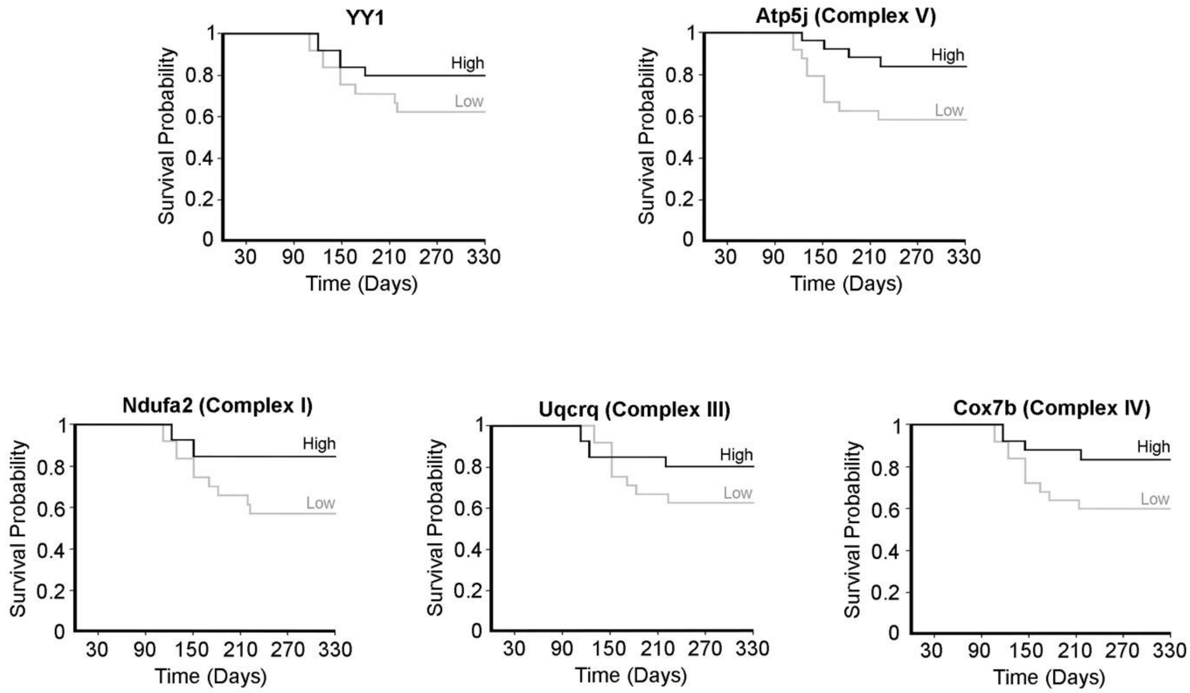

Supplementary Figure 7

Supplementary Fig.7| YY1 target genes found upregulated in eIF6 mutant mice show a trend of poor prognosis in primary liver cancer

All data were retrieved from PRECOG (https://precog.stanford.edu/) typing the official gene name.

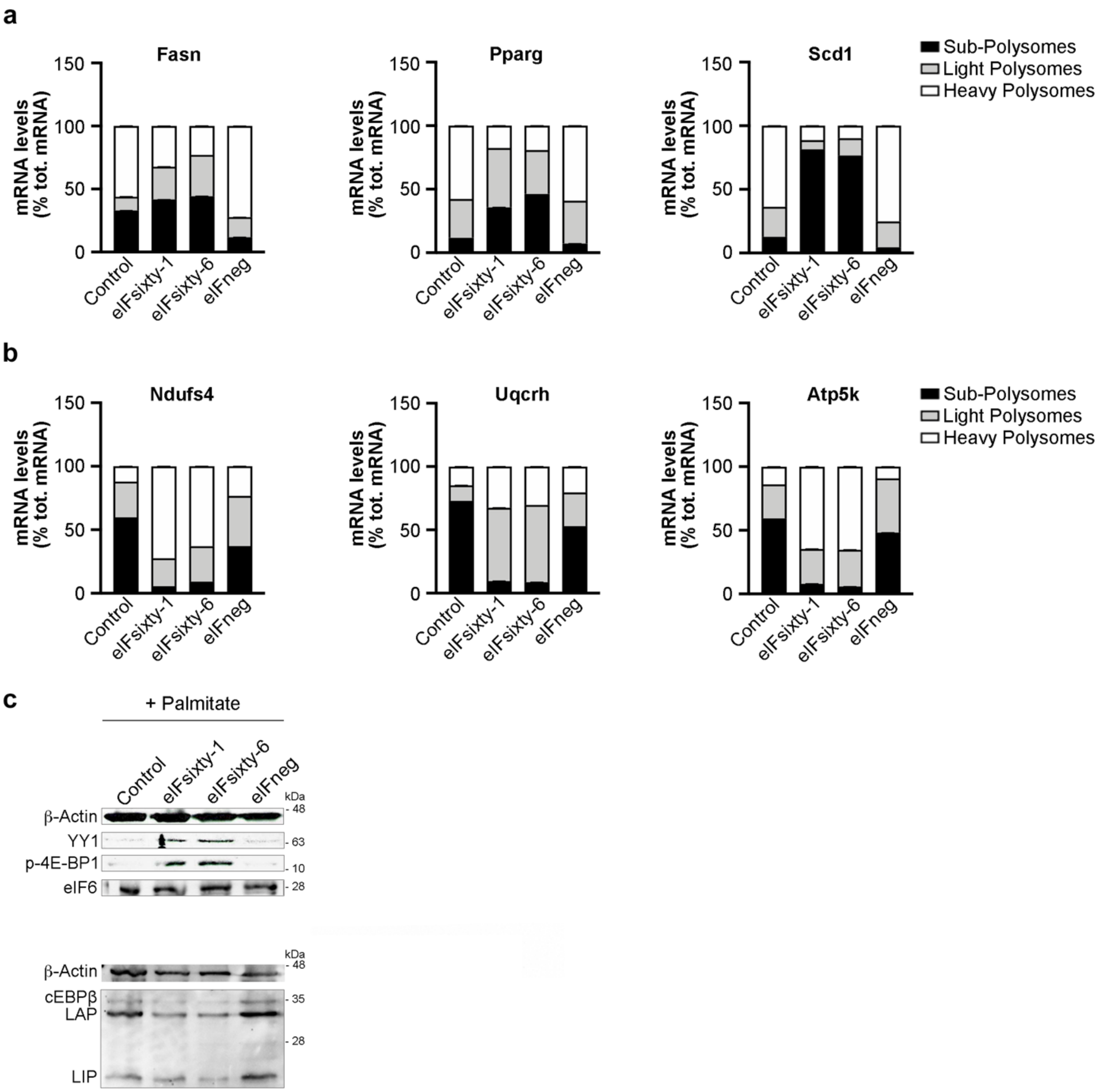

Supplementary Figure 8

Supplementary Fig.8| eIFsixty compounds affect the translation of lipogenic and mitochondrial mRNAs

a,b, Stacked bar charts represent the quantification of selected mRNA levels in heavy, light and subpolysomes of AML12 cells after administration of Palmitate and eIFsixty-i compounds. eIFneg was used as negative control. Real Time PCR analysis was performed on n=3 independent experiments. SD are less than 1%. **c**, Representative Western Blotting at indicated experimental conditions. eIFsixty-1 and eIFsixty-6 maintain 4E-BP1 phosphorylation and YY1 protein level. In contrast, C/EBPβ translation products are reduced in AML12 treated with eIFsixty-1 and eIFsixty-6 compounds. Data are provided as a Source Data File.

Supplementary Table 1. eIF6 mRNA is overexpressed during HCC progression and in metastasis.

Murine model	GEO Profile Accession number	eIF6 mRNA expression		P value
		No tumor (mean ± SD)	HCC (mean ± SD)	
Mdr2 KO	GDS1990	396,06 ± 61,89	609,35 ± 86,44	0,000305
Trim24 KO	GDS3087	698,70 ± 83,47	1038,52 ± 331,08	0,052807
Txnip deficient	GDS2006	254,51 ± 22,26	386,01 ± 40,60	0,000019

Supplementary Table 2. List of common genes between human HCC biomarkers (Diagnostic and Prognostic) and differential expressed genes in eIF6 heterozygous mice (editable: Supplementary Data Files under the section Source Data, Excel File, Human Data).

Genes Up-regulated in human HCC and Down-regulated in eif6+/- mice

Biomarker	Biomolecule	Subject	Regulation	Biomarker.s.t	Experiment	Level.of.signi	Source	PMID	log2FoldChange (eIF6+/- vs eIF6+/+ mice)
PDCD6IP	RNAs	Human	Differentially expressed between HCC and non-	Diagnostic	HCC v/s non-t	p < 0.01	Tissue	14642619	-0,505957879
RPS6KA3	RNAs	Human	Diffrenetially expressed in HCC v/s non-tumor	Diagnostic	HCC v/s non-t	p < 0.01	Tissue	11973655	-0,517559989
GLUL	RNAs	Human	Upregulated in HCV related HCC than normal c	Diagnostic	HCV related H	p < 0.01	Tissue	19821982	-0,574321688
GOLGA4	RNAs	Human	Upregulated in HCV related HCC than HCV rela	Diagnostic	HCV related H	p < 0.01	Tissue	19821982	-0,576646297
PARP1	RNAs	Human	Upregulated in tumor	Diagnostic	HCC v/s non-t	P<0.003	Tissue	16703398	-0,588531
ITGA9	RNAs	Human	Upregulated in PT than PN (with 3 fold change	Diagnostic	Metastatic HC	p < 0.01	Tissue	12640447	-0,596009022
ARHGEF12	RNAs	Human	Differentially expressed between HCC and norr	Diagnostic	HCC v/s norm	p < 0.01	Tissue	14642619	-0,644732644
PSMD1	RNAs	Human	Upregulated in HCC than non-tumorous tissue	Diagnostic	HCC v/s non-t	p < 0.05	Tissue	15756260	-0,715248079
ABCC3	RNAs	Human	overexpressed in hepatocellular carcinoma	Potential Diag	Normal vs HC	p < 0.01	Tissue	26337276	-0,723544142
PRPF8	RNAs	Human	Differentially expressed between HCC and non-	Diagnostic	HCC v/s non-t	p < 0.01	Tissue	14642619	-0,739058663
MYADM	RNAs	Human	Upregulated in HCC than non-tumor (with > 3	Diagnostic	HCC v/s non-t	p < 0.05	Tissue	18715028	-0,747206589
AOX1	RNAs	Human	Diffrenetially expressed in moderately differen	Diagnostic	moderately d	p < 0.01	Tissue	11973655	-0,751177226
LGALS1	RNAs	Human	upregulated in	Diagnostic an	Single nodula	p < 0.05	Tissue	16788756	-0,770892204
ROBO1	RNAs	Human	Upregulated (1.5 fold)	Diagnostic	HCC v/s Norm	p < 0.05 (for p	Tissue	28977866	-0,869983632
ITGB2	RNAs	Human	upregulated in	Diagnostic an	Single nodula	p < 0.05	Tissue	16788756	-0,873180589
COL4A1	RNAs	Human	Upregulated in HCC than non-tumor (with > 3	Diagnostic	HCC v/s non-t	p < 0.05	Cell line, Tissu	1917104	-1,003482229
RAD23A	RNAs	Human	Diffrenetially expressed in HCC v/s non-tumor	Diagnostic	HCC v/s non-t	p < 0.01	Tissue	11973655	-1,019143677
RFX5	RNAs	Human	Differentially Expressed in HCC Tumors than nc	Diagnostic	HCC v/s non-t	p < 0.01	Tissue	15057898	-1,024941128
COL15A1	RNAs	Human	Upregulated (1.5 fold)	Diagnostic	HCC v/s Norm	p < 0.05	Tissue	28977866	-1,100913022
BASP1	RNAs	Human	Upregulated in PT than PN (with 3 fold change	Diagnostic	Metastatic HC	p < 0.01	Tissue	12640447	-1,109381451
TOP2A	RNAs	Human	Upregulated (1.5 fold)	Diagnostic	HCC v/s Norm	p < 0.05	Tissue	28977866	-1,362032024
TOP2A	RNAs	Human	Upregulated in dedifferentiated HCC (with fold	Diagnostic	moderately ar	FDR < 2%.	Tissue	18820673	-1,362032024
CCND1	RNAs	Human	Differentially expressed between HCC and norr	Diagnostic	HCC v/s norm	p < 0.01	Tissue	14642619	-1,638133948
LIMK1	RNAs	Human	Upregulated in PT than PN (with 3 fold change	Diagnostic	Metastatic HC	p < 0.01	Tissue	12640447	-1,645830694
LCN2	RNAs	Human	Diffrenetially expressed in HCC v/s non-tumor	Diagnostic	HCC v/s non-t	p < 0.01	Tissue	11973655	-2,546599293
LCN2	RNAs	Human	Upregulated in HCC than non-tumorous tissue	Diagnostic	HCC v/s non-t	p < 0.05	Tissue	15735714	-2,546599293

Genes Up-regulated in human HCC and Down-regulated in eif6+/- mice

Biomarker	Biomolecule	Subject	Regulation	Biomarker.s.t	Experiment	Level.of.signi	Source	PMID	log2FoldChange (eIF6+/- vs eIF6+/+ mice)
ADNP	RNAs	Human	Upregulated in G2 than G1 (with fisher ratio m	Prognostic	G1 (well differ	P < 0.021	Tissue	15710396	-3,959626122
GOLGA4	RNAs	Human	Upregulated in G2 than G1 (with fisher ratio m	Prognostic	G1 (well differ	P < 0.017	Tissue	15710396	-0,576646297
ALPL	RNAs	Human	Discriminating among late-stage sample (betw	Prognostic	Edmondson g	p < 0.001	Tissue	16175600	-0,603939758
TBCE	RNAs	Human	Upregulated in G1 than L1 (with fisher ratio m	Prognostic	non-tumorou	P = 0.001	Tissue	15710396	-0,638568312
EIF4G1	RNAs	Human	Upregulated in G2 than G1 (with fisher ratio m	Prognostic	G1 (well differ	P < 0.007	Tissue	15710396	-0,660812248
MAN2B1	RNAs	Human	Differentially expressed gene	Prognostic	cirrhotic liver	p < 0.01	Tissue	19861515	-0,711587829
STK39	RNAs	Human	Upregulated in T3 than T1 and adjacent non-tu	Prognostic	Intrahepatic r	p < 0.05	Tissue	18504433	-0,732982526
GALNT10	RNAs	Human	Discriminating among late-stage sample (betw	Prognostic	Edmondson g	p < 0.001	Tissue	16175600	-0,742520489
FBN1	RNAs	Human	11 genes (FUT3, RDBP, E48, SEMA3F, COL6A3,	Prognostic	Intrahepatic r	p < 0.05	Tissue	15688398	-0,744024979
ALDH3A2	RNAs	Human	Differentially expressed gene	Prognostic	cirrhotic liver	p < 0.01	Tissue	19861515	-0,797288783
ROBO1	RNAs	Human	Upregulated in T3 than T1 and adjacent non-tu	Prognostic	Intrahepatic r	p < 0.05	Tissue	18504433	-0,869983632
SOX4	RNAs	Human	Upregulated in T3 than T1 and adjacent non-tu	Prognostic	Intrahepatic r	p < 0.05	Tissue	18504433	-0,925476008
CORO1C	RNAs	Human	Upregulated in liver cancer tissues of HCCLM9-	Prognostic	Agressive or a	p < 0.05	Tissue and Cel	20181269	-0,962059208
RFX5	RNAs	Human	Discriminating among late-stage sample (betw	Prognostic	Edmondson g	p < 0.001	Tissue	16175600	-1,024941128
TNFAIP3	RNAs	Human	13 genes (FUT3, RDBP, E48, SEMA3F, COL6A3,	Prognostic	Intrahepatic r	p < 0.05	Tissue	15688398	-1,176252778
MBD4	RNAs	Human	Upregulated in late cirrhosis than early cirrhos	Prognostic	Early v/s Late	p < 0.05	Tissue	15108252	-1,266679019
ANXA2	RNAs	Human	Genes associated with EHR of HCC	Prognostic	Extrahepatic r	P< 0.05	Tissue	17016605	-1,298436317
SAA1	RNAs	Human	Upregulated in late cirrhosis than early cirrhos	Prognostic	Early v/s Late	p < 0.05	Tissue	15108252	-1,505781698
SAA2	RNAs	Human	Upregulated in late cirrhosis than early cirrhos	Prognostic	Early v/s Late	p < 0.05	Tissue	15108252	-2,264324888
IQGAP3	RNAs	Human	Upregulated in highly invasive HCC than less-in	Prognostic	less (L) invasiv	p < 0.01	Tissue	19945130	-2,358576431
VCL	RNAs	Human	Upregulated in recurrence HCC than non-recur	Prognostic	Early recurren	p < 0.05	Tissue	17391314	-2,39952645
IPO9	RNAs	Human	Upregulated in highly invasive HCC than less-in	Prognostic	less (L) invasiv	p < 0.01	Tissue	19945130	-0,53160156
UACA	RNAs	Human	Discriminating among late-stage sample (betw	Prognostic	Edmondson g	p < 0.001	Tissue	16175600	-0,55027783
HK2	RNAs	Human	Differentially expressed (14 Upregulated and 4	Predictive	treatment res	p < 0.01	Tissue	15447987	-1,43803922

Genes Down-regulated in human HCC and up-regulated in eif6+/- mice

Biomarker	Biomolecule	Subject	Regulation	Biomarker.s.t	Experiment	Level.of.signi	Source	PMID	log2FoldChange (eIF6+/- vs eIF6+/+ mice)
ATP5J2	RNAs	Human	Downregulated in among all types HCC v/s non-tum	Diagnostic	HCC v/s non-t	p < 0.01	Tissue	16391793	1,386625847
NDUFA2	RNAs	Human	7 genes Downregulated i.e. CYP1A2, Human-alpha1-a	Diagnostic an	Single nodula	p < 0.05	Tissue	16788756	1,363916636
RPS17	RNAs	Human	Downregulated in among all types HCC v/s non-tum	Diagnostic	HCC v/s non-t	p < 0.01	Tissue	16391793	1,255796525
KLKB1	RNAs	Human	Downregulated in HCC than non-tumor (with fold ch	Diagnostic	HCC v/s non-t	p < 0.05	Tissue	16463013	1,106051616
NDUFS6	RNAs	Human	Downregulated in HCC than non-tumor	Diagnostic	HCC v/s non-t	p < 0.05	Tissue	11752456	0,920409713
CETN2	RNAs	Human	Downregulated in HCC than non-tumor	Diagnostic	HCC v/s non-t	p < 0.05	Tissue	11752456	0,876574552
NDUFA1	RNAs	Human	Downregulated in HCC than non-tumor	Diagnostic	HCC v/s non-t	p < 0.05	Tissue	11752456	0,817591814
SOD1	RNAs	Human	Downregulated in all three tested HCC samples.	Diagnostic	HCC v/s non-t	p < 0.07	Tissue	11813304	0,696972823
OTC	RNAs	Human	Downregulated in HCC than non-tumor (with > 3 fold	Diagnostic	HCC v/s non-t	p < 0.05	Tissue	18715028	0,688760099
CDO1	RNAs	Human	Downregulated in HCC than non-tumor	Diagnostic	HCC v/s non-t	p < 0.05	Tissue	11752456	0,625450036
QDPR	RNAs	Human	Downregulated in HCC than non-tumor	Diagnostic	HCC v/s non-t	p < 0.05	Tissue	11752456	0,62332922
NDUFS8	RNAs	Human	Downregulated in HCC than non-tumor	Diagnostic	HCC v/s non-t	p < 0.05	Tissue	11752456	0,577717244

Genes Down-regulated in human HCC and up-regulated in eif6+/- mice									
Biomarker	Biomolecule	Subject	Regulation	Biomarker.s.t	Experiment	Level.of.signi	Source	PMID	log2FoldChange (eIF6+/- vs eIF6+/+ mice)
MDK	RNAs	Human	Upregulated in High-risk individuals with cirrhosis v	Prognostic	High-risk indi	p < 0.0003	Tissue	14768006	1,625709691
STAT1	RNAs	Human	Downregulated in G2 than G1 (with fisher ratio more	Prognostic	G1 (well diffe	P < 0.012	Tissue	15710396	0,624460062
IGF1	RNAs	Human	Downregulated in late cirrhosis than early cirrhosis (	Prognostic	Early v/s Late	p < 0.05	Tissue	15108252	0,61850415
IFIT1	RNAs	Human	Downregulated in late cirrhosis than early cirrhosis (	Prognostic	Early v/s Late	p < 0.05	Tissue	15108252	0,576318676
KDELR2	RNAs	Human	Downregulated in G3 compared with G2 (with Fisher	Prognostic	G2 (moderate	P < 0.017	Tissue	15710396	0,558059773

Supplementary Table 3. Comparison of genes network profiles from DE genes derived from RNA-Seq data of eIF6$^{+/-}$ livers and severe NAFLD patients: key-genes that correlate to NAFLD/NASH progression into HCC are shown (editable: Supplementary Data Files under the section Source Data, Excel File, Human Data).

GENE	PROTEIN	Textmining FDR	p-value eIF6+/- mouse model	Most significant homolog mouse gene
PLIN2	perilipin 2	0,014307110	0,011663628	PLIN2
LPL	lipoprotein lipase	0,013584660	0,043906679	LPL
VLDLR	very low density lipoprotein receptor	0,019863960	0,017888211	VLDLR
CIDEC	cell death inducing DFFA like effector c	0,024664540	0,047603829	CIDEC
BCL2A1	BCL2 related protein A1	0,030371800	0,00012604	BCL2L13
CNBP	CCHC-type zinc finger nucleic acid binding protein	0,014307110	0,024780275	CNBP
SMPD2	sphingomyelin phosphodiesterase 2	0,013584660	5,13E-06	SMPD3
PPARA	peroxisome proliferator activated receptor alpha	0,013584660	0,0000779	PPARA
PPARGC1A	PPARG coactivator 1 alpha	0,042396190	0,004807679	PPARG
PLIN1	perilipin 1	0,014307110	0,07751956	PLIN1
CD36	CD36 molecule	0,013584660	0,008579742	CD36
FADS2	fatty acid desaturase 2	0,019163800	0,001922173	FADS2
PLEK	pleckstrin	0,030371800	0,000199365	PLEKHB2
IRS2	insulin receptor substrate 2	0,049564780	0,00000282	IRS1
SCD	stearoyl-CoA desaturase	0,013584660	0,003866425	SCD
TNF	tumor necrosis factor	0,030371800	2,35E-04	TNFRSF19
LIPA	lipase A, lysosomal acid type	0,020083730	0,049928495	LIPA
FGF21	fibroblast growth factor 21	0,018319170	0,004371208	FGF9
YWHAZ	tyrosine 3-monooxygenase/tryptophan 5monooxyg	0,013584660	0,348861984	YWHAZ
SLC27A4	solute carrier family 27 member 4	0,020753290	0,204987023	SLC27A4
BCL2A1D	BCL2 related protein A1D	0,013584660	0,162406064	BCL2A1D
CCL2	C-C motif chemokine ligand 2	0,042626840	0,117686005	CCL2
SREBF2	sterol regulatory element binding transcription facto	0,013584660	0,24176236	SREBF2
APP	amyloid beta precursor protein	0,039327400	0,175723457	APP
DGAT1	diacylglycerol O-acyltransferase 1	0,013584660	0,504858944	DGAT1
