# Supplementary material for: Targeting of eIF6-driven translation induces a metabolic rewiring that reduces NAFLD and the consequent evolution to hepatocellular carcinoma
Source: Nat Commun. 2021 Aug 12;12:4878. doi: 10.1038/s41467-021-25195-1 (PMC8361022; doi:10.1038/s41467-021-25195-1)
Supplement: Supplementary file 3 — Description of Additional Supplementary Files [file 41467_2021_25195_MOESM3_ESM.pdf]

Description of Additional Supplementary Files

Title: Supplementary Data 1

Description: Excel Tables for RNAseq data of HFD mice

Title: Supplementary Data 2

Description: Excel editable Tables for Supplementary Tables 2-3
