# Supplementary material for: Targeting of eIF6-driven translation induces a metabolic rewiring that reduces NAFLD and the consequent evolution to hepatocellular carcinoma
Source: Nat Commun. 2021 Aug 12;12:4878. doi: 10.1038/s41467-021-25195-1 (PMC8361022; doi:10.1038/s41467-021-25195-1)
Supplement: Supplementary file 6 — Reporting Summary [file 41467_2021_25195_MOESM6_ESM.pdf]

nature portfolio

Corresponding author(s): Biffo

Last updated by author(s): Jun 23, 2021

Reporting Summary

Nature Portfolio wishes to improve the reproducibility of the work that we publish. This form provides structure for consistency and transparency in reporting. For further information on Nature Portfolio policies, see our Editorial Policies and the Editorial Policy Checklist.

Statistics

For all statistical analyses, confirm that the following items are present in the figure legend, table legend, main text, or Methods section.

n/a	Confirmed	
☐	☒	The exact sample size (n) for each experimental group/condition, given as a discrete number and unit of measurement
☐	☒	A statement on whether measurements were taken from distinct samples or whether the same sample was measured repeatedly
☐	☒	The statistical test(s) used AND whether they are one- or two-sided<br>*Only common tests should be described solely by name; describe more complex techniques in the Methods section.*
☒	☐	A description of all covariates tested
☐	☒	A description of any assumptions or corrections, such as tests of normality and adjustment for multiple comparisons
☐	☒	A full description of the statistical parameters including central tendency (e.g. means) or other basic estimates (e.g. regression coefficient) AND variation (e.g. standard deviation) or associated estimates of uncertainty (e.g. confidence intervals)
☐	☒	For null hypothesis testing, the test statistic (e.g. F, t, r) with confidence intervals, effect sizes, degrees of freedom and P value noted<br>*Give P values as exact values whenever suitable.*
☒	☐	For Bayesian analysis, information on the choice of priors and Markov chain Monte Carlo settings
☒	☐	For hierarchical and complex designs, identification of the appropriate level for tests and full reporting of outcomes
☒	☐	Estimates of effect sizes (e.g. Cohen's d, Pearson's r), indicating how they were calculated

Our web collection on statistics for biologists contains articles on many of the points above.

Software and code

Policy information about availability of computer code

Data collection	R3.3.1; DESeq2; Prism8; Fiji (image); Microsoft excel
Data analysis	Analyses of human datasets was conducted using R 4.4.1 software. RNA-seq data analysis from eIF6 wt and het livers: Reads counts were normalized by calculating a slze factor, as implemented in DESeq2. Analyses were performed in R version 3.3.1.

For manuscripts utilizing custom algorithms or software that are central to the research but not yet described in published literature, software must be made available to editors and reviewers. We strongly encourage code deposition in a community repository (e.g. GitHub). See the Nature Portfolio guidelines for submitting code & software for further information.

Data

Policy information about availability of data

All manuscripts must include a data availability statement. This statement should provide the following information, where applicable:

- Accession codes, unique identifiers, or web links for publicly available datasets
- A description of any restrictions on data availability
- For clinical datasets or third party data, please ensure that the statement adheres to our policy

Data availability statement: All data supporting the findings of this study are provided within the paper and its Supplementary information. Source data files are provided with this paper. We confirm that all mouse strains used here are readily available from the authors (eIF6 knockout) or from EMMA mouse repository (EM:07840). Further information on research design is available in the Nature Research Reporting Summary linked to this article. Any data are available from the authors upon request. The RNAseq data generated in this study have been deposited in the www.ebi.ac.uk/arrayexpress/ database under accession code ID: E-MTAB-9009 [https://www.ebi.ac.uk/arrayexpress/experiments/E-MTAB-9009/]. Retrieved human data were from: GEO, https://www.ncbi.nlm.nih.gov/sites/GDSbrowser?acc=GDS4881 (32 NAFLD). For the 125 obese patients dataset, the ethical approval of the study does not allow to publicly share the patients data. All

data, code, and materials used in this last analysis are available upon reasonable request for collaborative studies regulated by materials/data transfer agreements (MTA/DTAs) to either biffo@ingm.org or directly to the original author, luca.valenti@unimi.it.

Field-specific reporting

Please select the one below that is the best fit for your research. If you are not sure, read the appropriate sections before making your selection.

☒ Life sciences ☐ Behavioural & social sciences ☐ Ecological, evolutionary & environmental sciences

For a reference copy of the document with all sections, see nature.com/documents/nr-reporting-summary-flat.pdf

Life sciences study design

All studies must disclose on these points even when the disclosure is negative.

Sample size	For animal studies, we did not predetermine sample size but we used group sizes on basis of previous experiments using similar methodologies. To minimize any potential biases, we randomly assigned mice of same genotype for all our studies.
Data exclusions	No samples or animals were excluded.
Replication	All experimental findings were reproduced as stated in figure legends. All additional replication attempts were successful.
Randomization	All samples were randomly allocated
Blinding	The experiments were not blinded. However, we followed standard laboratory procedures of randomization. Each experiment was associated with the proper controls, and compared samples were collected and analyzed under the same condition.

Reporting for specific materials, systems and methods

We require information from authors about some types of materials, experimental systems and methods used in many studies. Here, indicate whether each material, system or method listed is relevant to your study. If you are not sure if a list item applies to your research, read the appropriate section before selecting a response.

Materials & experimental systems

n/a	Involved in the study	
☐	☒	Antibodies
☐	☒	Eukaryotic cell lines
☒	☐	Palaeontology and archaeology
☐	☒	Animals and other organisms
☒	☐	Human research participants
☒	☐	Clinical data
☒	☐	Dual use research of concern

Methods

n/a	Involved in the study	
☒	☐	ChIP-seq
☒	☐	Flow cytometry
☒	☐	MRI-based neuroimaging

Antibodies

Antibodies used	Rabbit polyclonal anti-eIF6 (Biffo S. et al, 1997; 1:1000 for immunoblotting and 1: 200 for immunohistochemistry); Mouse monoclonal anti-b-Actin (Sigma-Aldrich, Cat#A5441, 1:4000 for immunoblotting); Mouse monoclonal anti-YY1(Santa Cruz Biotechology, Cat#sc-7341, 1:500 for immunoblotting); Rabbit monoclonal anti-phospho-(Ser235/236) -rpS6 (Cell Signaling, Cat#4858, 1:1000 for immunoblotting and 1: 200 for immunohistochemistry); Rabbit monoclonal anti-rpS6 (Cell Signaling, Cat#2217, 1:1000 for immunoblotting); Rabbit monoclonal anti-phospho-(Ser65)-4E-BP1 (Cell Signaling, Cat#9451, 1:1000 for immunoblotting); Rabbit monoclonal anti-4E-BP1 (Cell Signaling, Cat#9644, 1:1000 for immunoblotting); Mouse monoclonal anti-H-Ras (Santa Cruz Biotechology, Cat#sc-35, 1:1000 for immunoblotting); Purified anti-C/EBPb (Biolegend, cat#606202, 1:500 for immunoblotting); Rabbit monoclonal anti-Tomm20 (Cell Signaling, Cat#42406, 1:200 for immunofluorescence); Rabbit monoclonal anti-AIF (Cell Signaling, Cat#5318, 1:200 for immunofluorescence).
Validation	All antibodies sourced from commercial corporation are well validated by the manufacturer and are widely used in the scientific community for Western Blotting and Immunofluorescence.<br>For Rabbit polyclonal anti-eIF6 (Biffo S. et al, 1997; 1:1000 for immunoblotting and 1: 200 for immunohistochemistry)<br>Validated in our papers published before.<br><br>For Mouse monoclonal anti-b-Actin (Sigma-Aldrich, Cat#A5441, 1:4000 for immunoblotting) https://www.sigmaaldrich.com/catalog/product/sigma/a5441?lang=it&region=IT&gclid=EAIaIQobChMI76-9u5- V6gIVQ4uyCh08OA9GEAAYASAAEgIer_D_BwE<br>Validated by other users, cited 7501 times in the company website<br>For Mouse monoclonal anti-YY1(Santa Cruz Biotechology, Cat#sc-7341, 1:500 for immunoblotting) https://www.scbt.com/p/yy1-antibody-h-10<br>Validated by other users, cited 182 times in the company website

For Rabbit monoclonal anti-phosho-(Ser235/236) -rpS6 (Cell Signaling, Cat#4858, 1:1000 for immunoblotting and 1: 200 for immunohistochemistry)
https://www.cellsignal.co.uk/products/primary-antibodies/phospho-s6-ribosomal-protein-ser235-236-d57-2-2e-xp-rabbit-mab/4858
Validated by other users, cited 680 times in the company website

For Rabbit monoclonal anti-rpS6 (Cell Signaling, Cat#2217, 1:1000 for immunoblotting) https://www.cellsignal.com/products/primary-antibodies/s6-ribosomal-protein-5g10-rabbit-mab/2217 Validated by other users, cite 1143 times in the company website

For Rabbit monoclonal anti-phospho(Ser65)-4E-BP1 (Cell Signaling, Cat#9451, 1:1000 for immunoblotting) https://www.cellsignal.com/products/primary-antibodies/phospho-4e-bp1-ser65-antibody/9451 Validated by other users, cited 313 times in the company website

For Rabbit monoclonal anti-4E-BP1 (Cell Signaling, Cat#9644, 1:1000 for immunoblotting) https://www.cellsignal.com/products/primary-antibodies/4e-bp1-53h11-rabbit-mab/9644 Validated by other users, cited 572 times in the company website

For Mouse-Purified anti-C/EBP þ (3 isoforms C/EBP þ, LAP, LIP) Antibody (Biolegend, cat#606202, 1:500 for immunoblotting) https://www.biolegend.com/en-us/search-results/purified-anti-c-ebp-beta-3-isoforms-c-ebp-beta-lap-lipc-ebpbeta-c-ebp-beta-antibody-1984
Validated by other users, cited 2 times in the company website

For Mouse monoclonal anti-H-Ras (Santa Cruz Biotechology, Cat#sc-35, 1:1000 for immunoblotting) https://www.scbt.com/p/h-ras-antibody-c-20
Validated by other users, cited 103 times in the company website

For Rabbit monoclonal anti-Tomm20 (Cell Signaling, Cat#42406, 1:200 for immunofluorescence) https://www.cellsignal.com/products/primary-antibodies/tom20-d8t4n-rabbit-mab/42406 Validated by other users, cited 46 times in the company website

For Rabbit monoclonal anti-AIF (Cell Signaling, Cat#5318, 1:200 for immunofluorescence) https://www.cellsignal.com/products/primary-antibodies/aif-d39d2-xp-rabbit-mab/5318 Validated by other users, cited 80 times in the company website

Eukaryotic cell lines

Policy information about cell lines

Cell line source(s)	AML12 male mouse hepatocytes (ATCC, CRL-2254); Human embryonic kidney 293T (ATCC, CRL-3216); Mouse primary hepatocytes were isolated as described in this paper. Ear mesenchymal stem cell (EMSC) were isolated as previously described (Brina et al, 2015, doi:10.1038/ncomms9261)
Authentication	The cell lines have not been authenticated recently.
Mycoplasma contamination	The cell lines were tested negative for mycoplasma contamination.
Commonly misidentified lines (See ICLAC register)	No commercially misidentified cells were used.

Animals and other organisms

Policy information about studies involving animals; ARRIVE guidelines recommended for reporting animal research

Laboratory animals	eIF6+/+ and eIF6+/- transgenic mice were generated, backcrossed to C57BL/6N strain (Gandin V et al, 2008, doi:10.1038/nature07267. Epub 2008 Sep 10). A cohort of age-matched mice (n=12 for eIF6+/+ and n=15 for eIF6+/-) were fed with High-Fat Diet. For NAFLD/NASH/HCC mouse model, a cohort of age-matched mice (n=11 for eIF6+/+ and n=8 for eIF6+/-) were fed with High-Fat Diet plus high sugar drinking water and weekly injected i.p. with low doses of CCl4. For RNAseq data analysis four biological replicates for HFD-fed eIF6+/+ and three biological replicates for HFD-fed eIF6+/- mice were analyzed.
Wild animals	The study did not involve wild animals
Field-collected samples	The study did not involve field-collected samples
Ethics oversight	All mice were maintained under specific and opportunistic pathogen-free conditions and all experiments involving animals were performed in accordance with the Ethical Committee of San Raffaele by experimental protocols approved by national regulators (IACUC n.688). Mandatory rules for 4Rs and euthanasia were followed.

Note that full information on the approval of the study protocol must also be provided in the manuscript.
